# The development of musculoskeletal radiology for 100 years as presented in the pages of *Acta Radiologica*

**DOI:** 10.1177/02841851211050866

**Published:** 2021-10-19

**Authors:** Mats Geijer, Fatih Inci, Nektarios Solidakis, Pawel Szaro, Bariq Al-Amiry

**Affiliations:** 1 Department of Radiology, Institute of Clinical Sciences, Sahlgrenska Academy, 70712 University of Gothenburg, Gothenburg, Sweden; 2 Department of Radiology, Region Västra Götaland, 56749 Sahlgrenska University Hospital, Gothenburg, Sweden; 3 Department of Clinical Sciences, Lund University, Lund, Sweden; 4 Department of Clinical Science, Intervention and Technology, Karolinska Institute and Karolinska University Hospital, Stockholm, Sweden

## Abstract

During the last 100 years, musculoskeletal radiology has developed from bone-only radiography performed by everyone to a dedicated subspecialty, still secure in its origins in radiography but having expanded into all modalities of imaging. Like other subspecialties in radiology, it has become heavily dependent on cross-sectional and functional imaging, and musculoskeletal interventions play an important role in tumor diagnosis and treatment and in joint diseases. All these developments are reflected in the pages in *Acta Radiologica*, as shown in this review.

## Introduction

After Wilhelm Conrad Röntgen discovered the “X-strahlen” in late 1895, they were rapidly applied in medicine across the world. Alban Köhler had published his monograph on bone radiography in 1901 (1). He later published “Lexikon der Grenzen des Normalen und der Anfänge des pathologischen im Röntgenbilde” in 1910 (2), later concentrating on bone imaging and still in print (3). Many other examples of successful bone radiography were published around the turn of the century, e.g. Thor Stenbeck's monograph “Om röntgenstrålarne” in Swedish in 1900 (4). By 1921, when the first issue of *Acta Radiologica* was published, bone radiography had already reached certain maturity. Extremity radiography was of high quality, with specific radiographic projections described as early as 1905 (5). Technical advances in tube design also permitted radiography of thicker body parts such as the spine or pelvis. Arthrography with air or oxygen had been demonstrated in 1905 and 1906, and conventional tomography had been described in 1914 and 1915, with the first workable patent applied for by Bocage in 1921 (6).

### Eponymous diseases

Several of the eponymous musculoskeletal diseases that we meet almost every day were first described in *Acta Radiologica*. In 1929, Hans Jessen Panner (7) described “a peculiar affection of the capitulum humeri…” (8–10), today still known as Panner's disease. Christian Ingerslev Baastrup (11), in several articles, described inflammation between the lumbar spinous processes (12,13), the “kissing spine” disease, or eponymous Baastrup's disease. Sinding-Larsen-Johansson disease (14) was independently reported by Christian Magnus Falsen Sinding-Larsen in *Acta Radiologica* in 1921 (15) and by Sven Johansson in 1922 (16). Even later, in times of more developed radiography and medicine, were new eponymous diseases described in *Acta Radiologica*, such as van Buchem's disease in 1955 (17) and the similar Ribbing's disease (18), described by Seved Ribbing in 1949 (19).

### Techniques

#### Tomography

Tomography was an early technique to improve on projection radiography, which was discovered in “five countries by at least nine investigators” (6). However, it was difficult to apply the principles in practice. One of the first pioneers to develop a working apparatus was Bernard Ziedses des Plantes, who in 1931 presented his thesis (20) and one year later reported on the technique in *Acta Radiologica*, calling it planigraphy (21). The technique became widely used in all fields of radiography not least in musculoskeletal imaging, and the technical aspects were further perused in some papers in *Acta Radiologica* (22,23). Its use for spondylodiscitis and in trauma was reported in 1949 (24). With the arrival of computed tomography (CT), the use of the technique rapidly declined. With the introduction of digital radiography at the beginning of the new millennium, there was renewed interest in tomography, now as digital linear tomography, however turning out to be most useful in chest (25,26) and breast (27) imaging.

#### Arthrography

In *Acta Radiologica*, two papers report on the use of pneumoarthrography in the knee, including a case series of lipoma arborescens (28,29). Arthrography with a positive contrast medium did not come into more general use until later in the 1930s, when the development of urographic contrast media permitted comparatively painless examinations without the risk of air embolism associated with the insufflation of large amounts of air. Lindblom early reported on arthrography of the knee and shoulder in 1938 and 1939 (30,31), after which followed many reports on arthrography of the knee and shoulder, and also the ankle (32), elbow (33), and wrist (34). Much effort was put in to improve the arthrographic technique to visualize the menisci better (35–37). Conventional arthrography of the hips was mostly concerned with Legg-Calvé-Perthes disease in children (38,39).

With the advent of CT and magnetic resonance imaging (MRI), arthrography of the knee became almost non-existent, whereas, from 1996, other areas such as the shoulder with MRI (40,41) or CT (42), the wrist (43), and hip (44,45) became more prominent research areas. Indirect MR arthrography of the shoulder was reported by Van Dyck et al. (46) in 2009.

#### Angiography

The modern angiographic technique with catheter replacement was introduced by Sven-Ivar Seldinger in 1953 (47). Angiography in the musculoskeletal field was first reported in musculoskeletal tumor imaging. Bartley and Wickbom (48) in 1959 reported on angiography in soft-tissue hemangiomas, which was followed by a few other reports by other authors. In the 1970s, there was an interest in angiography in extremity trauma (49,50) and in 1982, a comparison with CT was published (51). With improved angiographic techniques, there was a renewed interest in tumor imaging at the end of the 20th century (52**)** and angiography migrated from conventional catheter techniques towards CT (53,54) and MR (55**)** angiography in tumor evaluation.

#### Interventional techniques

Interventional needle-based techniques have been performed using radiography, CT, ultrasound, and MRI; the choice of imaging modality depends on the type of structure to biopsy or inject and the anatomical location. Personal preference and local factors also may play a role. Mostly, a biopsy has been performed for tumor diagnosis or follow-up (see further under the “Tumors” section) but also for infection and inflammation. The use of needle biopsy in the spine was described in a review article in 2007 (56). Articles about therapeutic interventions have mainly dealt with vertebroplasty and kyphoplasty (57) from 2004 onwards. Not only biopsy techniques and results but also new positioning devices and biopsy needles have been introduced in *Acta Radiologica*, both in its beginning (58) and in later years (59,60).

#### Nuclear medicine techniques

Although beta, positron, and gamma emitters had been tried earlier, it was not until technetium-99 m (^99m^Tc)-labeled phosphates or diphosphonates were introduced that bone scintigraphy became clinically useful (61,62). The development of the imaging technology (63,64) with, for example, pinhole collimators further improved image quality. Bone scintigraphy was initially reported for facial and skull imaging, not least by Bergstedt and co-workers (65), who in seven articles in 1975–1981 described in detail bone scintigraphy of the facial skeleton. In the following years, different ^99m^Tc compounds were compared (66), and imaging of occult fractures became one of the main applications of bone scintigraphy (67–69). The possibility for whole-body imaging, coupled with the high sensitivity for bone remodeling, made bone scintigraphy an excellent modality for staging bone involvement in malignant disease, reported in several papers (70–72). The scintigraphic appearance of Paget's disease of bone was reported in 1990 (73,74), the latter article also reporting the use of single-photon emission CT (SPECT) (74). Bone scintigraphy was also reported in the assessment of children with Legg-Calvé-Perthes disease (39), and chronic recurrent multifocal osteomyelitis (75). After the introduction of MRI, it was compared with bone scintigraphy for bone stress injury (76,77). Fusion imaging with planar bone scintigraphy and radiographs was reported for clinical scaphoid fracture (78). The development of nuclear medicine continued with the introduction of positron emission tomography (PET)/CT, which for musculoskeletal imaging has its primary focus in oncologic imaging and metastasis screening (79,80), also for skeletal muscle metastasis imaging (81). PET/CT also has an important role in imaging of infection, both for spinal infection (82) and in imaging infected prostheses (83).

#### Computed tomography

The introduction of whole-body CT in the late 1970s was a revolution in all fields of musculoskeletal imaging, reflected in the growing number of articles in *Acta Radiologica*. The first musculoskeletal article was on vertebral rotation in scoliosis by Aaro et al. (84) in 1978 using an early whole-body CT, something very difficult to perform with radiography. Similarly, tibial torsion was measured with CT in a couple of articles using an EMI scanner (85) and a whole-body scanner, concluding that no CT method of measurement was clinically useful (86). During the first decade, this was followed by articles on measurement of bone mineral content in osteoporosis (87–89) and diagnosis and evaluation of musculoskeletal tumors (51,90,91). Anda et al. (92) reported on the new sector angle measurement in adult acetabular dysplasia in 1986. In degenerative disease, CT was reported for meniscal evaluation in 1984 (93) and shoulder arthroplasty in 1987 (94). Spinal imaging was reported for diagnosis and postoperative imaging of low back pain and cervical spine spondylosis, comparing it with myelography and MRI (95–97). Bone trauma imaging was not at the forefront during the early years due to the missing possibility to perform multiplanar reformations (MPR), which is so important today. To overcome this obstacle, Muren et al. (98) published a report on CT in scaphoid trauma, imaging the scaphoid in the long axis by the use of a special stand in 1990. CT diagnosis of all aspects of musculoskeletal diseases would increase dramatically over the following decades, and CT is today a workhorse of all musculoskeletal imaging. Low-dose musculoskeletal CT, achieving an effective dose comparable to that of conventional radiography of the same organ, has been reported in several papers for lumbar spine imaging (99–101) and the pelvis and hip (102,103) between 2014 and 2019.

Further developments of CT have included dual-energy CT (DECT), in musculoskeletal imaging used for prosthesis imaging with its potential for metal artifact reduction (104,105), for imaging of gout, and trauma imaging, using the potential for calcium subtraction to visualize bone marrow edema (106,107) Cone-beam CT (CBCT), first introduced for dental imaging using a flat-panel detector, has been used for extremity CT in a variety of applications such as scaphoid trauma (108), rheumatoid arthritis (109), and foot imaging under weight-bearing (110), and in *Acta Radiologica* reported for evaluation of knee arthroplasty in 2018 (111), showing high potential for determining the rotation of femoral and tibial component loosening.

#### Ultrasonography

One of the first articles on ultrasound of Baker's cysts was published in *Acta Radiologica* as early as 1980 (112). Ultrasound equipment did not, however, become of sufficiently high quality for musculoskeletal applications until the mid-1990s. Before 1990, only a few reports on muscle hematoma in hemophiliacs (113), the hand in rheumatoid arthritis (114), the rotator cuff (115), hip joint synovitis in children (116), and osteochondritis dissecans in the knee (117) were published. Other interesting reports were published in 1990 by Myllymäki et al. (118) on ultrasound of jumper's knee, in 1995 by Höglund et al. (119) about the dislocated ulnar collateral ligament in the Stener lesion of the thumb, and in 1997 Finnbogason and Jorulf (120) described the method for dynamic evaluation of the unstable infant hip by ultrasound, still used in most pediatric ultrasound examinations for suspected hip instability.

Ultrasound, like CT, has expanded its possibilities with improved hardware and software. Its applications today are numerous in fields such as tendon imaging in the shoulder (121) and ankle (122), biopsy guidance, and peripheral arthritis diagnosis and follow-up (123), often performed by rheumatologists.

#### Magnetic resonance imaging

Musculoskeletal MRI was first reported by Pettersson et al. (124) in 1985, on MRI of sacrococcygeal tumors, also summarizing musculoskeletal MRI of the day in the following issue of *Acta Radiologica* (125). MRI was quickly adopted as an important imaging modality for musculoskeletal use, and as the image quality improved and imaging time decreased, the number of articles in *Acta Radiologica* rapidly increased (126). For example, spinal imaging was first reported in 1987 (127), hemophilic arthropathy in 1987 (128), a comparison of low-field MRI with scintimetry for evaluation of post-traumatic femoral head avascular necrosis (129), and an evaluation of soft-tissue infection by ultra-low-field MRI in 1989 (130), and the use of gadolinium contrast for investigation of soft-tissue tumors in 1990 (131). Today, the ubiquitous use of MRI is evident in the large proportion of the published articles in *Acta Radiologica*.

### Infection

Tuberculosis was of great clinical concern when *Acta Radiologica* started. The treatment options at that time were light and radiation treatment for lupus vulgaris and other superficial lesions, and various forms of surgery for deeper-seated lesions, until streptomycin became available in 1945 (132). For the first two decades, many papers were published on various aspects of tuberculosis, also in the musculoskeletal system. Already, in the first volume, Collin reported on tuberculosis of the joints and its treatment with light baths (133). Other reports followed, also on the influence of tuberculosis on the growth in children (134). Then, as now, tuberculous spondylitis was of interest and Westermark argued for the use of oblique images (135). After World War II, in parallel with the introduction of the first antibiotics, the publication of tuberculosis-related articles waned. In the 1990s, there was a renewed interest in tuberculosis, again on spinal tuberculosis diagnosis, now using MRI (136) and on the drainage of associated psoas abscesses (137). Drainage of psoas abscesses was described also for pyogenic infection (138,139). In 1996, *Acta Radiologica* published a series of five studies on different manifestations of tuberculosis, two of them on the musculoskeletal system (140,141). The focus of imaging has in later years mostly been on advanced imaging such as MRI (142,143) and PET/CT (144), also in *Acta Radiologica*. Perhaps more importantly, Andronikou et al. (145) described the imaging appearances of tuberculosis also on low-end modalities, which may be the only imaging option in low-income countries. In the same issue, section editor Seppo Koskinen in an editorial (146) stressed the importance of also diagnosing the comparatively rare manifestations of tuberculosis in the musculoskeletal system to make possible a rapid start of treatment of the infection.

The changes in focus on different infectious agents are reflected in the papers in *Acta Radiologica*. Radiologic changes from diseases that are today rare in high-income countries were reported early on, such as tetanus in 1938 (147), syphilis in 1945 (148), leprosy in 1950–1952 (149–151), and poliomyelitis in 1957 (152). The publications in later years have mostly focused on the ability of newer modalities to diagnose and follow up an infection before and after treatment (83,130,153–155), especially after spinal surgery (156,157), with some reports also on parasitic diseases (158–161).

### Tumors

In the beginning, *Acta Radiologica* was dedicated to both diagnostic and therapeutic radiology, not least of tumors, until the separation into a diagnostic *Acta Radiologica: Diagnosis* and *Acta Radiologica: Therapy, Physics, Biology* in 1963, eventually getting the name *Acta Oncologica* in 1987. Musculoskeletal tumors have been and still are one of the fundamental subjects in *Acta Radiologica* since the early publications. For example, Bichel and Kirketerp (162) wrote about myeloma in 1938, sarcomas were presented by Jönsson in 1939 (163), and a chordoma in a thoracic vertebra was described in 1941 (164).

Different modalities have different uses in the diagnosis and follow-up of tumors. Angiography became highly important after Seldinger reported his new catheter replacement technique (47). With the introduction of CT and MRI and the possibilities for CT and MR angiography, the need for conventional angiography waned. Today, one of the main modalities is CT. Many articles outlining the benefits of this modality were published from the very beginning of the era of CT. Argin et al. (53) in 1987 evaluated the effectiveness of CT angiography in determining vascular invasion in patients with musculoskeletal tumors. The development in CT and the decrease in radiation dose have been followed in *Acta Radiologica*.

One of the cornerstone modalities in musculoskeletal tumor imaging is MRI. It has demonstrated an important role in the diagnosis and staging of tumors before treatment and in the post-treatment phase. One of the first articles was written in 1983 by Volle et al. (165) comparing CT and MRI in bony lesions of the skull base. MRI was superior in defining the extensions of soft-tissue infiltration and arterial encasement. Jenner et al. (166) in 1996 wrote about the characteristic vascular fibrofatty structure on MRI in skeletal muscle hemangiomas. In a series of articles, Einarsdottir et al. (167–169) explored the use of MRI in the diagnosis of lipomatous tumors. Using intravenous gadolinium contrast added to the possibility to separate vessels from tumor-like lesions (165). In 1993, Søvik et al. (170) published an article about the changes in the pelvic wall after radiation treatment, reporting that the changes disappear more than a year after radiation therapy. Throughout the years of publications in *Acta Radiologica*, significant changes occurred in the understanding and development of MR sequences and their applications in clinical practice. Nouh et al. (171) in 2017 evaluated the potential of diagnostic imaging to identify and characterize the appendicular non-acral soft-tissue sarcomas with emphasis on their morphology on MRI.

Ultrasound also has an important role in the diagnosis of musculoskeletal tumors. One of the first published articles was in 1937 about the action of ultrasound waves on living tissue with special regard to the application for tumor treatment by van Everdingen (172). Musculo-skeletal ultrasound reached maturity in the 1990s and, like for CT and MRI, ultrasound devices and techniques developed rapidly, for instance, introducing the use of navigation technique (59). Rahmani et al. (173) in 2017 showed that ultrasound diagnosis of superficial lipomas has good sensitivity and specificity. Park et al. (174) in 2018 proved that the tumor border and peritumoral stroma obtained by shear wave elastography is stiffer compared with benign masses.

In later years, a considerable number of articles have assessed the aspects of positron emission tomography/CT (PET/CT) in tumor diagnosis and follow-up. In 1987, Surov et al. (81) published an article about PET/CT imaging of skeletal muscle metastases. Hsu et al. (175) in 2008 discussed the fluorodeoxyglucose (18F) (18F-FDG) uptake in brown tumors that mimics multiple skeletal metastases in primary hyperparathyroidism. Chang et al. (176) in 2014 found that 18F-FDG PET/CT is an accurate examination to detect skeletal metastases and is also superior to bone scintigraphy. Fiz et al. (177) in 2017 wrote about bony metastasis of prostate cancer, reporting that the degree of bone invasion and trabecular bone uptake are predictors of subsequent bone marrow failure before treatment in patients with significant bone tumor burden. Surov et al. (178) in 2020 reported the correlation between tumor hypoxia and 18F-FDG PET uptake.

Several articles in *Acta Radiologica* have described interventional procedures dealing with tumor diagnosis, biopsy, and treatment. Bone biopsy became a generally applicable method, first with high-quality fluoroscopy such as of the vertebrae in 1971 (179) and later with guidance by ultrasound (180), CT (181), and MRI. In 1993, Tikkakoski et al. (180) published an article about the combination of fine needle biopsy and cutting needle in percutaneous ultrasound-guided biopsy. The previous year, CT-guided bone biopsy in bone lesions had been found to be a safe, reliable, and cost-efficient method (181). Tehranzadeh et al. (56) in 2007 in a review article showed that CT-guided spinal biopsy is a safe, effective procedure and the procedure of choice in the definitive diagnosis of pathologic lesions of the spine. The accuracy of CT-guided spinal biopsy was further reported in 2011 (182).

#### Trauma

Most early publications on fracture diagnosis were by necessity limited to the peripheral extremities due to, by today's standards, the primitive equipment. The first publication about trauma was by Baastrup (183) in the third issue of *Acta Radiologica* in 1921 about the differentiation of os vesalianum and a proximal fracture in the fifth metatarsal bone. Stress fracture of the metatarsal bones was described by Runström (184) in 1924 and a form of a stress reaction in the tibia and femur by Hansson (185) in 1938. Nordentoft (186) in 1940 further reported on the similarity between stress fractures in unusual locations and the importance of not classifying them as bone sarcomas. With the introduction of scintigraphic and MRI methods at the end of the 20th century, stress fracture was further evaluated by bone scintigraphy and MRI (76,77). Myhre (187) in 1939 was one of the first to describe the radiologic appearance of osteochondral lesions of the talar trochlea. It took several decades from Röntgen's discovery in 1895 until trauma radiography also incorporated evaluation of the soft tissues. Lipohemarthrosis after knee trauma had been known by trauma surgeons for a long time but radiologically it was described for the first time in *Acta Radiologica*, in 1942 (188), demonstrating the need for obtaining the lateral knee radiograph after trauma with horizontal X-rays. Similarly, the observation that effusion after trauma in the pediatric elbow could be seen by observation of the fat pads on the lateral image was first reported in *Acta Radiologica*, in 1954 (189).

One of the significant problems in trauma management has been hip fractures, which were nearly impossible to treat and often constituted a death sentence until antisepsis, antibiotics, and appropriate osteosynthesis materials were introduced. The Smith-Peterson nail became available in 1925 and was refined by Sven Johansson (of the Sinding-Larsen-Johansson disease), reporting his findings in 1932 (190). With improved chances for a successful outcome for the patients, research in hip fracture diagnosis and classification increased. Between 1946 and 1988, nine papers dealt with diagnosis and evaluation of hip fractures, from a case series of femoral neck fractures after irradiation of cancer of the uterus (191) to an evaluation of three different osteosynthesis methods for trochanteric fractures (192). The diagnosis of hip fractures was an early musculoskeletal application for MRI, both for low-field MR scanners in 1989 (129) and high-field scanners in 1997 (193). CT was for a long time of insufficient quality to be reliable for evaluation of suspected occult hip fracture, but with multidetector CT (MDCT) and modern PACS systems with capabilities for multiplanar reformations (MPR), CT caught up with MRI in diagnostic accuracy (194), especially if using softer reconstruction algorithms to detect bone marrow edema using soft-tissue windowing (195).

The other focus area in bone trauma has been the diagnosis of scaphoid fracture. The abysmal outcome of a non-healed scaphoid fracture in a scaphoid nonunion advanced collapse (SNAC) wrist is obvious today, but it was first in 1937 that a specific radiographic projection for the scaphoid bone was described (196). In *Acta Radiologica*, 11 papers have dealt with different imaging modalities for scaphoid fracture evaluation; from radiography, first mentioned in 1949 (197), to bone scintigraphy in 1988 (67), CT in 1992 (198), and MRI in 1999 (199). The possibility of using digital radiography in areas demanding high resolution was shown in an analysis of digital scaphoid radiography in 1996 (200).

In extremity trauma, CT has become a workhorse for fracture evaluation, evident by the many papers published from 1992 (198) onwards. However, it was first with MDCT and the possibility of MPR in arbitrary planes that CT from 2004 was useful in extremity trauma (201–204). DECT is useful for bone marrow evaluation in trauma (107). MRI has become an invaluable tool for assessing suspected occult fractures (194), mostly in the scaphoid and hip, and for assessing all forms of soft-tissue trauma. Already in 1991, Tervonen et al. (205) reported on post-traumatic bone bruise in the knee, followed by numerous reports on cartilage, meniscal, and ligamentous injury from trauma. For shoulder trauma, Rand et al. (206) reported on the use of STIR images in 1998 followed by a large number of articles on trauma to the rotator cuff and labrum. A large number of articles dealt with the diagnosis and technique of shoulder arthrography from 1996 onward (40). Using MRI in trauma to the wrist was first reported in 1999 (199) and the elbow in 2005 (207).

### Derangement of joints

Overuse or post-traumatic changes in the joints have been studied and reported during all of *Acta Radiologica*'s existence. Besides conventional radiography, arthrography was the first technique that could be used to visualize the internal structures of the joints. Arthrography of the cruciate ligaments was described and evaluated by Lindblom in 1938 (30) and the radiographic findings in meniscal lesions were described in 1940 (208). Lindblom (36) later published his findings on knee arthrography as a Supplementum to *Acta Radiologica*. Lindblom (31) also described the technique and findings on shoulder arthrography. Although CT arthrography was a theoretical possibility, it was not really until the introduction of MRI that the internal joint structures could be studied either as non-enhanced MRI or as MR arthrography. Knee MRI began to be reported in the early 1990s (209,210), shoulder MRI in the late 1990s (206,211), and the Achilles tendon at the same time (212,213). MR arthrography of the shoulder was first reported in 1996 (40). CT arthrography with high-resolution MPR became possible somewhat later, reported for the shoulder in 2008 (42).

### Arthritis and inflammatory diseases

The first issue of *Acta Radiologica* in 1921 contained a case report of psoriatic arthritis (214). The early symptoms and healing phenomena in chronic rheumatic arthritis were described in 1943 (215). van Ebbenhorst Tengbergen and Dekkers (216) discussed Röntgen treatment of ankylosing spondylitis in 1941 and Overgaard (217) discussed the radiographic diagnosis in 1945. The unifying concept of spondyloarthritis was developed in the 1960s, embracing the different conditions ankylosing spondylitis, psoriatic arthritis, reactive arthritis, and enteropathic spondylitis. Sacroiliitis has been said to be the hallmark of ankylosing spondylitis, and the higher diagnostic ability of CT over radiography in diagnosing sacroiliitis was explored in a series of papers during 1998–2007 (218–220). A comparison between radiography, CT, and MRI was published in 2003 (221). The investigation of the often occult but unstable spinal fractures in ankylosing spondylitis was reported in two articles in 2004 (222,223). Hemophilic arthropathy was also described in the first issue of *Acta Radiologica* in 1921 (224) and more recently, MRI of hemophilic arthropathy has been investigated in several articles (128,225), also showing that gadolinium contrast is of limited value for evaluating synovial hypertrophy (225). Mustakallio reported the radiologic treatment of calcific tendinitis in the shoulder in 1939 (226). The uncovertebral joints and their degeneration were described in 1940 (227) and early symptoms and healing phenomena in rheumatoid arthritis in 1943 (215). Ochronosis was described a few years later (228). With the introduction of nuclear medicine and cross-sectional imaging, a new field of investigation opened. Evaluation of arthritis with MRI and intravenous contrast enhancement enabled the visualization of synovial inflammation in addition to a three-dimensional assessment of erosions (229,230). CT (218) and CBCT (109) facilitated the evaluation of bony changes. Ultrasound (114) of peripheral arthritis became available as a point-of-care modality, moving from the radiology department to the rheumatology department, where it has become an integral part of the clinical examination of the patient (231).

In cartilage and osteoarthritis imaging, Sven Ahlbäck (232) was the first to examine the knee joint in osteoarthritis in weight-bearing and created the Ahlbäck grading system for knee osteoarthritis. He published his findings in a Supplement to *Acta Radiologica* in 1968. With MRI, new methods for cartilage evaluation appeared, including dGEMRIC (233) and T2* mapping (234). The correlation between bone marrow edema and osteoarthritis was reported in 2008 (235).

## Discussion

The current review is by no means complete and in perusing the many articles published over 100 years, it is impressive both how much and rapidly radiology has evolved, and how interesting and informative even “old” articles can be. The articles published in *Acta Radiologica* over 100 years reflect not only the increased knowledge in medicine but also the changes in disease spectra, and the changes that the introduction of new imaging modalities have introduced in the possibility to diagnose and treat diseases and conditions that previously were more or less unreachable. *Acta Radiologica* will continue to inform its readers on the future development of musculoskeletal radiology for many years to come.
